# Diagnosing 12 prostate needle cores within an hour of biopsy via open-top light-sheet microscopy

**DOI:** 10.1117/1.JBO.25.12.126502

**Published:** 2020-12-15

**Authors:** Weisi Xie, Adam K. Glaser, Funda Vakar-Lopez, Jonathan L. Wright, Nicholas P. Reder, Jonathan T. C. Liu, Lawrence D. True

**Affiliations:** a University of Washington, Department of Mechanical Engineering, Seattle, Washington, United States; b University of Washington, Department of Laboratory Medicine and Pathology, Seattle, Washington, United States; c University of Washington, Department of Urology, Seattle, Washington, United States; d University of Washington, Department of Bioengineering, Seattle, Washington, United States

**Keywords:** prostate, cancer, diagnosis, biopsy, open-top light-sheet microscopy

## Abstract

**Significance:** Processing and diagnosing a set of 12 prostate biopsies using conventional histology methods typically take at least one day. A rapid and accurate process performed while the patient is still on-site could significantly improve the patient’s quality of life.

**Aim:** We develop and assess the feasibility of a one-hour-to-diagnosis (1Hr2Dx) method for processing and providing a preliminary diagnosis of a set of 12 prostate biopsies.

**Approach:** We developed a fluorescence staining, optical clearing, and 3D open-top light-sheet microscopy workflow to enable 12 prostate needle core biopsies to be processed and diagnosed within an hour of receipt. We analyzed 44 biopsies by the 1Hr2Dx method, which does not consume tissue. The biopsies were then processed for routine, slide-based 2D histology. Three pathologists independently evaluated the 3D 1Hr2Dx and 2D slide-based datasets in a blinded, randomized fashion. Turnaround times were recorded, and the accuracy of our method was compared with gold-standard slide-based histology.

**Results:** The average turnaround time for tissue processing, imaging, and diagnosis was 44.5 min. The sensitivity and specificity of 1Hr2Dx in diagnosing cancer were both >90%.

**Conclusions:** The 1Hr2Dx method has the potential to improve patient care by providing an accurate preliminary diagnosis within an hour of biopsy.

## 1 Introduction

Prostate cancer is the most common cancer of men in the United States. Approximately 191,930 new cases of prostate cancer will be diagnosed in 2020, and 33,330 men will die of prostate cancer.^1^ To diagnose prostate cancer, standard practice is to obtain 12 needle core biopsies of the prostate. The biopsy tissue is fixed in formalin, dehydrated, and embedded in paraffin, from which thin sections are cut, stained with hematoxylin and eosin dyes (H&E), and diagnosed by a pathologist. This procedure typically takes at least a day, in which there is a high degree of anxiety for most patients while awaiting the diagnosis.^2^^,^^3^ If cancer is diagnosed, a follow-up appointment is made to discuss treatment options. Making this appointment could require days or weeks of additional time. If it were technically possible to obtain a rapid diagnosis immediately after biopsy, while the patient is still on-site, a same-day treatment decision could be made, thus streamlining patient care. Rapid examination of 12-core biopsies, or MRI-targeted biopsies, could also streamline the selection of tumor-enriched biopsy cores for molecular analyses.

Faster histopathology procedures do exist, such as “rush” processing of biopsies in a 4-h, labor-intensive process.^4^ Alternatively, frozen sections can be prepared and diagnosed for each biopsy, a procedure that could take up to 2 h for all 12 biopsies. However, both of these procedures can compromise the quality of histology and, consequently, diagnostic accuracy.^4^^–^^6^ In addition, these procedures, which involve cutting thin sections of tissue, consume valuable biopsy material, which can compromise subsequent molecular assays.

To address these shortcomings, we developed a nondestructive tissue-processing and 3D microscopy method to enable pathologists to rapidly diagnose a set of 12 prostate needle cores. This time-efficient workflow, which we refer to as one-hour-to-diagnosis (1Hr2Dx), is nondestructive, making the tissues fully available for downstream histology and molecular assays, e.g., immunohistochemistry (IHC) and sequencing. Furthermore, 1Hr2Dx provides volumetric imaging data that increases tissue sampling, which is a potential advantage over conventional “gold-standard” histology. This study is an assessment of the ability of pathologists to diagnose a set of 12 needle core biopsies within an hour of receipt with high diagnostic accuracy.

## 2 Materials and Methods

### 2.1 Collection and Processing of Prostate Biopsies

We obtained from the IRB-approved biorepository at the University of Washington 44 needle core biopsy specimens that had been collected *ex vivo* from 7 radical prostatectomies using an 18-gauge (approximately 1-mm inner diameter) needle biopsy device (BARD^®^MAX-CORE^®^, Bard Biopsy, Tempe, Arizona). These biopsies were fixed in an aqueous solution of phosphate-buffered formalin at room temperature for 24 h, prior to commencing the 1Hr2Dx procedure. Each biopsy was deidentified and tracked with a biopsy ID. Additional fresh biopsies were also handled by the 1Hr2Dx procedure to assess the robustness of the procedure using unfixed tissue.

**Fig. 1:**
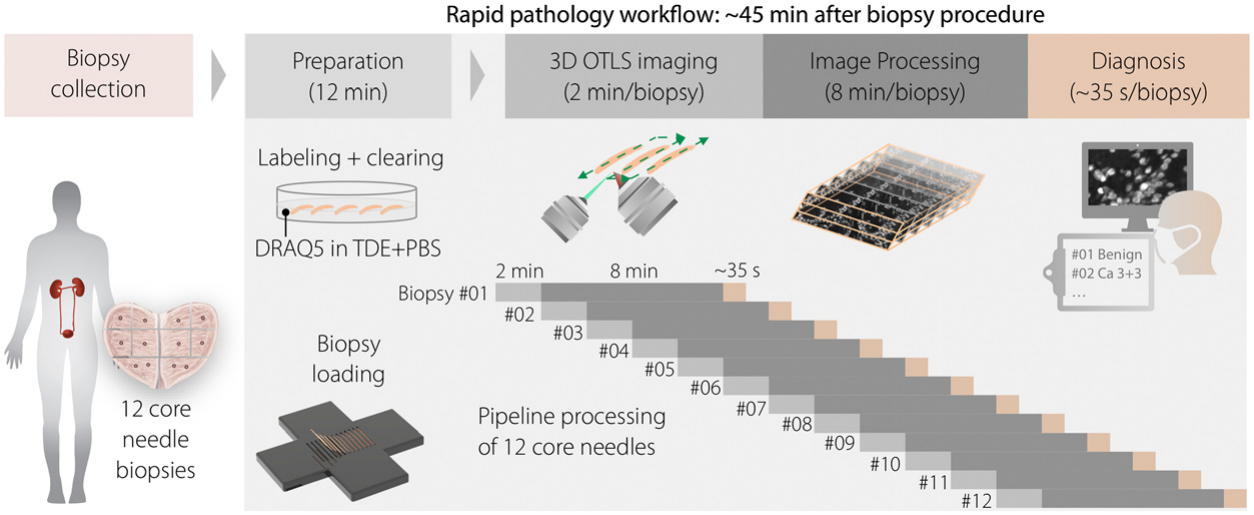
Sequence of the 1Hr2Dx workflow. The 12 biopsies are simultaneously cleared and labeled with the nucleic-acid-targeted fluorophore DRAQ5. The biopsies are then loaded onto a custom 12-biopsy sample holder and imaged in 3D with an OTLS microscope to a depth of ∼80  μm. Imaging (2  min/biopsy) and image processing (8  min/biopsy) of each biopsy occur sequentially. The pathologist views images of each biopsy as the datasets become available (Video 1, MP4, 33.3 MB [URL: https://doi.org/10.1117/1.JBO.25.12.126502.1]).

We performed the staining and clearing for each biopsy in separate wells in a multi-well plate, in which biopsy IDs were tracked. Biopsies were incubated in a solution of 0.1 mM nucleic-acid binding fluorophore DRAQ5 (ThermoFisher, catalog No. 62251) in a mixture of 2,2′-thiodiethanol (TDE, Sigma-Aldrich, CAS No. 111-48-8) and PBS buffer at room temperature for 5 min. The TDE/PBS ratio was adjusted to achieve a refractive index of 1.46, which matches that of the fused silica plate used as the sample stage holder on our open-top light-sheet (OTLS) microscope.^7^ This 5-min staining/clearing procedure provided sufficient optical clearing to allow us to image the biopsies to a depth of ≤100  μm. After staining/clearing, the biopsies were rinsed 3× in the TDE+PBS buffer (30 s per rinse) and imaged by OTLS microscopy (see Sec. 2.3). To minimize turnaround time, we clarified and labeled all 12 biopsies simultaneously and loaded them onto the microscope using a custom-made 12-biopsy holder (see Fig. 1). We imaged all biopsies sequentially and tracked the biopsy IDs for each 3D imaging dataset. Sets of 12 biopsies were imaged and diagnosed in a pipeline manner (Fig. 1). In brief, as each biopsy was imaged and became available as a 3D dataset, a pathologist could immediately view and diagnose the biopsy, panning through the range of depths at different magnifications (Video 1). Subsequent biopsies were continuously imaged and made available for diagnosis.

After imaging by 1Hr2Dx, all biopsies were formalin-fixed and paraffin-embedded (FFPE) for standard slide-based H&E histology. Immunohistochemical stains for keratin 5 (Cell Marque, clone 34BE12, at titer 1:2000) and keratin 8 (Cell Marque, clone 35BH11, at titer 1:100) were also performed on tissue sections from the biopsies.

### 2.2 Biopsy Holder

A custom sample holder, consisting of a 6-mm-thick Delrin plate with 12 biopsy-sized slots machined on one side of the plate, was designed for this study (see Fig. 1). Each slot measured 0.9 mm (width) × 0.6 mm (depth) × 2.5 cm (length). Each slot was made only 0.6-mm deep to allow the ∼1  mm in diameter biopsies to be slightly flattened/compressed against the transparent sample stage for time-efficient 3D imaging.

### 2.3 Open-Top Light-Sheet Microscopy

We have published many of the technical details of the multi-immersion OTLS microscope used in this study, including specifications for the computational hardware.^7^ The following features and settings are specific for this project. The nucleic-acid-targeting fluorophore, DRAQ5, is excited by a 638-nm laser (Cobolt Skyra™, Cobolt AB, Solna, Sweden). Emission fluorescence is collected by a multi-immersion objective (#54–10–12, Special Optics, Applied Scientific Instrumentation). The fluorescence is filtered with a bandpass filter (FF01–721/65–25, Semrock, IDEX Health & Science, LLC, Rochester, New York) and imaged onto a high-speed 2048×2048-pixel sCMOS camera (ORCA-Flash4.0 V2, Hamamatsu, Japan), which is capable of generating data at 800  MB/s. This objective and camera combination produces a field of view of ∼0.9  mm (width of light-sheet image) with near-Nyquist sampling of ∼0.44  μm/pixel. The vertical field of view is reduced to ∼80  μm to match the depth of focus of the illumination light sheet, which results in a 2048×256-pixel region of interest on the camera. For this study, the camera was operated with 2× binning to decrease both imaging time and dataset size, resulting in a sampling pitch of ∼0.9  μm/pixel and an overall resolution of ∼1.8  μm. By adjusting the stage-scanning speed, the spatial interval between successive frames is chosen as ∼0.62  μm to match the lateral sampling pitch with 2× camera binning. This level of resolution is roughly equivalent to the resolution provided by a 10× objective on a standard wide-field light microscope and allows the datasets to be both post-processed and visualized as grayscale images within an hour.

### 2.4 Imaging, Data Processing, and Visualization

Raw 2D light-sheet images are captured at a 45-deg angle with respect to the specimen surface and are serially acquired along the stage-scanning direction to scan a 0.9-mm-wide volumetric image “strip.” It is worth noting that the 0.9-mm width of this image strip matches the width of the biopsy specimens, which are loaded into rectangular channels that are aligned with the primary scanning direction of the OTLS microscope. Multiple biopsies are thus imaged sequentially based on the preset coordinates that are entered into the imaging code. For image processing, the images are sheared by 45 deg to represent their correct geometrical relationship in the 3D volume.^7^ The OTLS images are then sharpened with a Python-based edge-enhancement algorithm (based on Sobel edge detection).^8^ Finally, the images are re-saved into the XML/HDF5 file format, which allows them to be quickly opened and viewed at various depths and levels of magnifications using the ImageJ BigStitcher/BigDataViewer plug-in.^9^ With this plug-in, 3D images are first opened in a low-resolution zoomed-out view to obtain an overview of the whole biopsy. A pathologist can pan through different depths (within a range of 0 to 80  μm) of the biopsy (Video 1). When suspicious or ambiguous regions are identified, users can then view the regions at a higher resolution.

The images are acquired and processed on a workstation PC and streamed to a local server with direct-attached network storage. The pathologists review the images from the local server, which is independent of the acquisition workstation, so the image-viewing program (i.e., ImageJ) is not slowed down by the consumption of computational resources on the acquisition PC during imaging and processing.

The 12 biopsies are imaged, processed, and diagnosed in a pipeline manner, as shown in Fig. 1. After OTLS imaging of each biopsy is completed, post-processing for that biopsy immediately begins while subsequent biopsies are imaged in continuous succession. Once a biopsy dataset is fully processed and re-saved as a 3D image, a pathologist can immediately begin to view and diagnose the images from that 3D dataset. This pipeline workflow makes it possible to obtain a preliminary diagnosis of a set of 12 biopsies within an hour of biopsy collection.

### 2.5 Pathology Diagnosis

Three genitourinary pathologists independently reviewed both the OTLS-generated grayscale fluorescence images (nuclear staining only) and the H&E images from the FFPE tissue in a randomized order, categorizing each core as “cancer” or “benign,” based on their best judgment. Different identifiers were used for the two histology modalities to mask correspondence between the 1Hr2Dx and H&E images. In our experiment, the 1Hr2Dx pipeline shown in Fig. 1 was performed up through the stage of image processing. The pathologists reviewed all of the processed biopsies later, recording their turnaround time for diagnosing each biopsy. In a clinical setting, if a pathologist is available to interpret images as soon as they are generated, there would be no additional delays in terms of computational or file-access times. According to the timeline shown in Fig. 1, if one assumes that a pathologist is available to diagnose the biopsy images as soon as they are generated, the total turnaround time (staining/clearing, biopsy loading, imaging, post-processing, and diagnosis) for each set of 12 biopsies is dominated by the tissue-processing and imaging time (44 min) plus the short time it takes to diagnose the final biopsy in the set (35 s). To establish a ground truth for biopsies that were ambiguous based on standard H&E histology, we immunohistochemically stained these biopsies with anti-keratin antibodies (CK5/CK8). Those atypical glands that lacked a basal cell layer (evidenced by absence of a keratin 5 immunoreactive cell layer) were categorized as “cancer.” This use of keratin immunolabeling (when needed) to assist with the diagnosis of prostate cancer is standard pathology practice.^10^

### 2.6 Statistical Analysis

To calculate the true positive, true negative, false positive, and false negative values for the 1Hr2Dx diagnoses, we regarded the diagnoses of H&E-stained sections, supplemented if necessary with keratin IHC, as the “ground truth” based on the majority opinion of the three pathologists involved in this study. Sensitivity, specificity, accuracy, and positive and negative predictive values were determined for each pathologist as well as for the majority opinion of the pathologists in their diagnosis of the 1Hr2Dx images. Inter-rater agreements for both modalities of tissue processing—1Hr2Dx and H&E histology—were assessed by the Randolph’s free-marginal multi-rater kappa.^11^

## 3 Results

### 3.1 OTLS Images of Prostate Biopsies

**Fig. 2:**
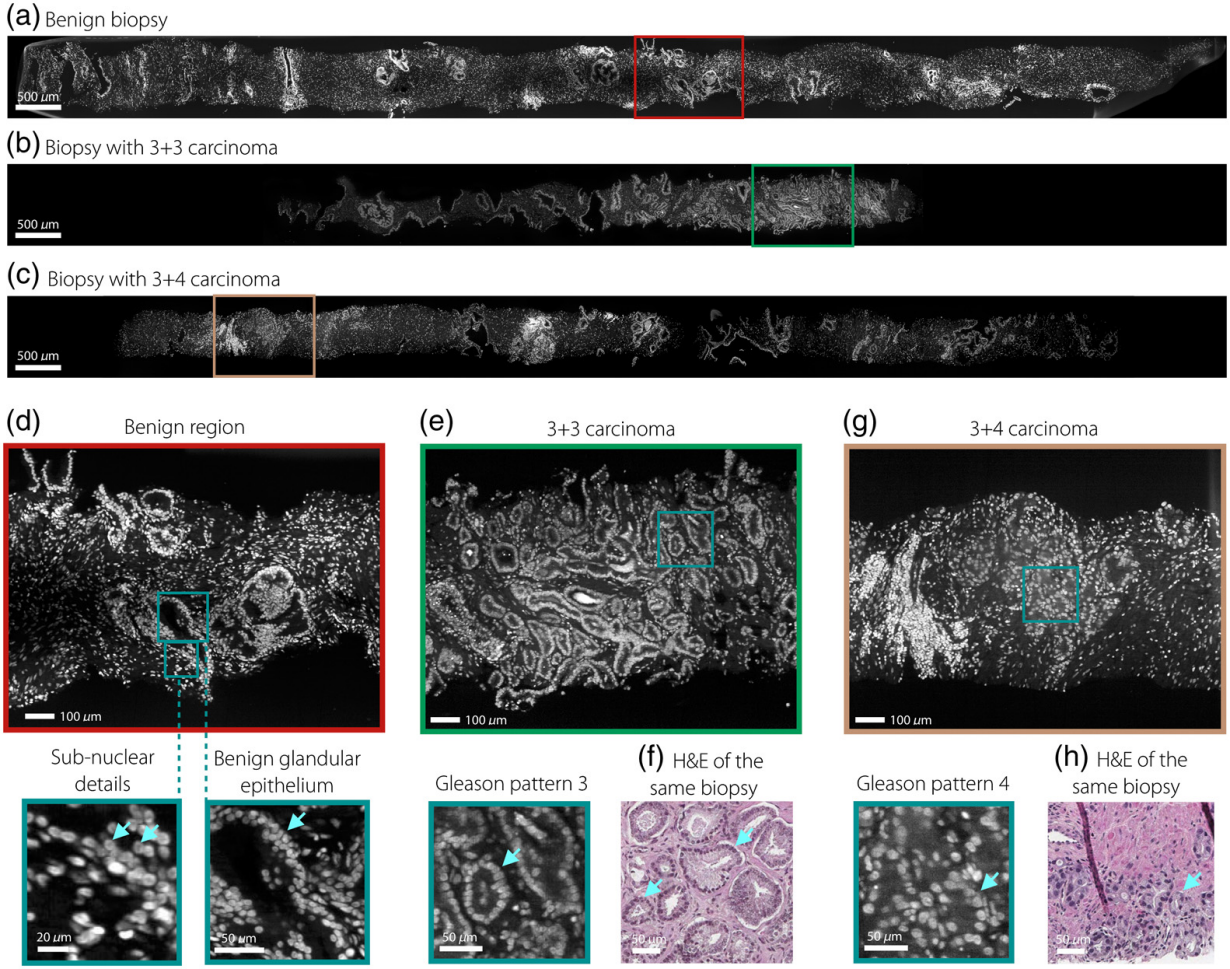
Representative 1Hr2Dx images of three biopsies showing regions of (a) benign prostate tissue, (b) Gleason score 3+3 adenocarcinoma, and (c) Gleason score 3+4 adenocarcinoma. Higher-magnification views of these three biopsies show (d) benign glands with the 2-cell layer of epithelial cells, which is characteristic of benign glands (arrow), (e) well-formed cancer glands that are characteristic of Gleason pattern 3 carcinoma (arrow), and (g) fused glands that are characteristic of Gleason pattern 4 carcinoma (arrow). H&E images of (f) Gleason pattern 3 carcinoma and (h) Gleason pattern 4 carcinoma are paired with corresponding 1Hr2Dx images.

Images of three ∼2-cm-long biopsies produced by our 1Hr2Dx platform are shown in Fig. 2, which demonstrates that structures of diagnostic importance are clearly seen.

**Fig. 3:**
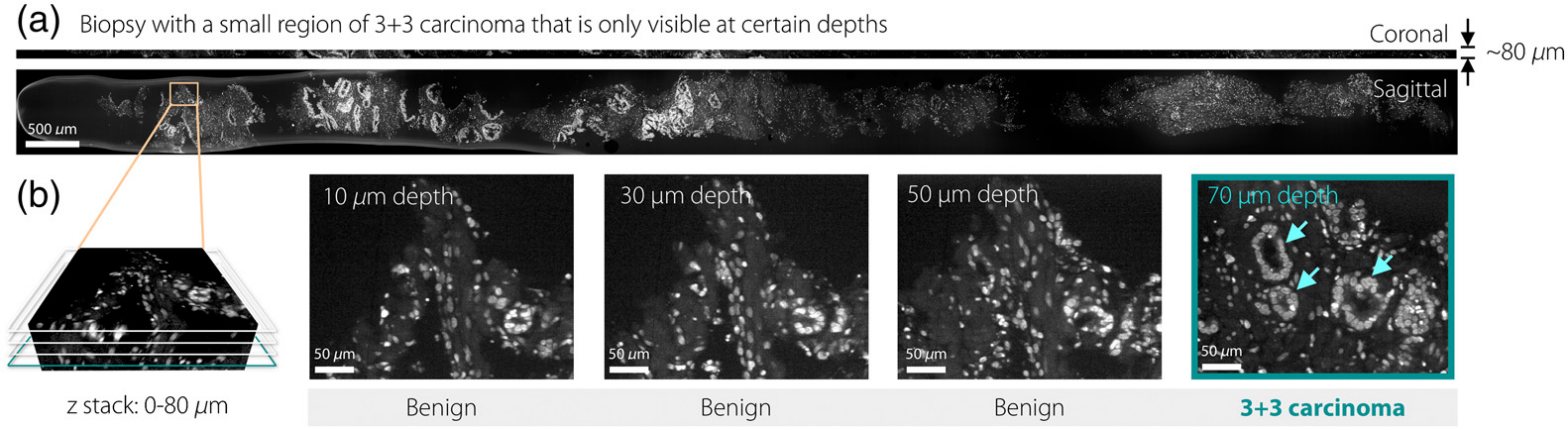
1Hr2Dx images of a biopsy in which a focus of adenocarcinoma is seen only at the 70-μm image level (arrow).

To illustrate the importance of increased sampling, images of a prostate biopsy with Gleason score 3+3 adenocarcinoma are shown (Fig. 3). It is worth noting that the small focus of carcinoma is only seen clearly in the image plane at a depth of 70  μm, whereas the other image depths reveal only benign glands. Thus, it is likely that this case would have received a false-negative diagnosis if only a few 2D histology sections had been viewed.

In addition to imaging formalin-fixed biopsies, we imaged fresh unfixed biopsies and verified that image qualities were comparable to fixed tissue using our 1Hr2Dx method (Fig. S1 in the Supplementary Material).

### 3.2 Pathology Analysis

Ten of the 44 biopsy cores contained cancer based on conventional H&E and IHC images of FFPE tissue. Accuracy, sensitivity, specificity, and positive and negative predictive values were determined for each pathologist and for the majority opinion of the three pathologists in their diagnosis of the 1Hr2Dx images. The diagnosis based on standard slide-based histology (H&E staining supplemented, if needed, with CK5/CK8 IHC) of the same biopsies was considered the ground truth. In general, the 1Hr2Dx diagnoses made by the three pathologists agreed with diagnoses based on conventional histology; the majority-opinion diagnoses yielded >0.9 accuracy, sensitivity, specificity, and negative predictive value (Table 1). The positive predictive value was lower at 0.82, presumably due to the increased sampling of our 3D pathology methods. For example, in one case, cancer was seen in the 1Hr2Dx images, but not in the slide-based H&E sections. Of the three pathologists, pathologists 1 and 2 were more accurate in their diagnoses, having an accuracy of 0.93 and 0.91, respectively. Pathologist 3, who had less experience with our 3D pathology technologies, made more false-positive diagnoses, resulting in lower accuracy, positive predictive value, and specificity (Table 1). We assessed the inter-rater agreements of diagnosis for each processing method (1Hr2Dx and H&E) using percentage overall agreement and Randolph’s free-marginal kappa (Table 2). Although the three pathologists agreed in their diagnoses of all slide-based H&E histology images, their agreement in diagnoses of 1Hr2Dx images was lower—82% overall (0.64 kappa value).

**Table 1:** Performance of three pathologists in diagnosing the 1Hr2Dx images. All 1Hr2Dx performance metrics were >0.9 except for a slightly lower positive predictive value, which is attributed to the increased sampling of our 3D pathology methods (i.e., increased tumor-detection sensitivity) compared with gold-standard slide-based histology.

	Pathologist 1	Pathologist 2	Pathologist 3	Majority diagnosis
Accuracy	0.93	0.91	0.77	**0.93**
Sensitivity	0.90	0.60	0.90	**0.90**
Specificity	0.94	1.00	0.74	**0.94**
Positive predictive value	0.82	1.00	0.50	**0.82**
Negative predictive value	0.97	0.89	0.96	**0.97**

**Table 2:** Inter-rater agreement of the three pathologists assessed by percent agreement overall and by the Randolph’s free-marginal multi-rater kappa.

	OTLS	H&E
Percent overall agreement	81.82%	100.00%
Free-marginal kappa	0.64	1
95% CI for free-marginal kappa	[0.46, 0.81]	[1.00, 1.00]

Of the majority-opinion 1Hr2Dx diagnoses, two were “false-positive” diagnoses and one was a “false-negative” diagnosis. The three pathologists reviewed these discrepancies between OTLS and H&E images retrospectively and independently. In false-positive case #1, the small cluster of atypical glands originally interpreted as cancer was re-interpreted as atrophy, as confirmed by H&E and IHC of this biopsy. For false-positive case #2, volumetric imaging revealed a malignant region that was not sampled by the H&E section. In other words, 1Hr2Dx provided superior sampling of the biopsy and therefore greater sensitivity for detecting cancer than slide-based pathology. Interestingly, the false-negative case also resulted from a sampling difference since the range of tissue imaged by 1Hr2Dx did not appear to include the cancer region that was seen in the H&E section.

The average turnaround time for the three pathologists to view the 1Hr2Dx images and diagnose each biopsy was 35 s (range 29 to 47 s). The average time from biopsy retrieval to diagnosis of a set of 12 needle core biopsies was 44.5 min. Based on visual examination, our protocol did not appear to compromise the quality of downstream H&E histology or IHC of formalin-fixed biopsies. Immunoreactivity of tissue for anti-keratin antibodies did not appear to be affected by 1Hr2Dx for fixed or fresh biopsies. The OTLS image quality of fixed and fresh specimens was comparable (Fig. S1 in the Supplementary Material).

## 4 Discussion

Providing a rapid diagnosis of prostate needle core biopsies plays an important clinical role both in quality of life for patients and for optimizing clinical outcomes through appropriate and timely treatments. In routine pathology practice, sets of 12 prostate needle core biopsies from each patient are batch processed with all specimens received in the pathology laboratory on a given day. H&E-stained sections of these biopsies are available no sooner than the day following receipt of the biopsies. Immunohistochemical stains, which may be performed to clarify the nature of the glands, typically require at least one additional day.

We developed and assessed a 1Hr2Dx procedure that would enable patients to obtain a preliminary diagnosis on-site after their biopsy procedures. In terms of turnaround time, we found that both fixed and fresh biopsies could be stained/clearing, imaged with 3D OTLS microscopy, post-processed (3D datasets), and interpreted by pathologists within an hour (average time of 44.5 min). The 1Hr2Dx procedure did not significantly impact the quality of downstream slide-based H&E histology or the immunoreactivity of standard keratins (IHC).

In terms of the diagnostic accuracy of 1Hr2Dx, the majority-opinion diagnosis of the three pathologists yielded >0.9 accuracy, sensitivity, specificity, and negative predictive value. The positive predictive value for 1Hr2Dx was 0.82, which we attribute to the increased sampling achieved by 1Hr2Dx. Certain discrepancies between pathologists, which were described in Sec. 3, could be attributed to pathologist 3 having less experience with OTLS images than pathologists 1 and 2. It is worth noting that, while 3D OTLS microscopy can be used to generate H&E-like color images of tissues stained with a fluorescent analog of H&E,^7^^,^^12^^–^^14^ this 1Hr2Dx study only yielded grayscale images of fluorescently labeled nuclei, for which there is a diagnostic learning curve. Also, it is worth noting that all 3 pathologists agreed in their diagnoses of slide-based H&E histology, for which they are all highly experienced. To provide more context on the characteristics of the biopsies in this study, in Table S1 in the Supplementary Material, we report the Gleason grade (majority opinion of three pathologists) for the 10 cancerous biopsies as well as the length of cancer with respect to the total biopsy length, all based on the 2D slide-based H&E histology images. It is worth noting that Gleason grading and cancer volume estimation were done retrospectively after we completed our pilot study to evaluate diagnostic accuracy.

Previous studies have reported the extent of inter-pathologist variance in diagnosing prostate cancer based on conventional slide-based histology.^15^^–^^17^ In one study, two pathologists independently reviewed biopsies from 34 patients and disagreed on the presence of cancer in 31 of 407 slides.^15^ Another study of 71 prostate biopsies reported similar levels of inter-pathologist variance (kappa 0.36 to 0.59).^16^ These studies, which were all based on routinely processed tissue, provide a goal and context for evaluating the performance of our platform.

We plan to improve our protocol and instrumentation to address several shortcomings. (1) Our 3D imaging was limited to ∼80  μm below the biopsy tissue surface. Given that our OTLS microscope is capable of comprehensively imaging entire 1-mm-diameter biopsies that are well-cleared,^7^^,^^14^ we will continue to optimize our staining/labeling protocols to further increase the 3D sampling extent of each specimen within the 1-h workflow. We expect that this will allow us to identify more small foci of cancer that are missed when relying upon 5-μm-thick histology sections, though this will also lead to longer diagnostic interpretation times. (2) We noted that in some fresh biopsies imaged with 1Hr2Dx, some epithelial cells detached from the stroma, as can be seen in subsequent slide-based histology images (Fig. S2 in the Supplementary Material). Introducing a brief formalin-fixation step prior to 1Hr2Dx or within the 1Hr2Dx procedure (so that the tissues are fixed throughout the 1Hr2Dx procedure) could prevent this artifact. (3) To reduce the learning curve for pathologists and to facilitate clinical adoption, we plan to implement a rapid fluorescent analog of H&E and modify the OTLS collection path for simultaneous dual-channel imaging,^13^ in conjunction with a rapid false-coloring method.^18^^,^^19^ (4) Since large 3D image datasets can be time-consuming to visualize and interpret, machine-learning techniques will be explored to segment critical structures,^20^^–^^23^ such as prostate glands, and to automatically identify a subset of localized regions of interest or ambiguity for pathologists to manually review, thus expediting and potentially improving the accuracy of pathologist review.^24^

Future scaled up studies will enable us to (1) further assess the diagnostic accuracy of our enhanced methods with a broad range of pathologists, (2) fully validate our ability to process and diagnose fresh biopsies with negligible degradation in tissue quality, and (3) assess the accuracy of pathologists to rapidly grade cancer.

Finally, studies will be needed in the future to determine how best to incorporate a 1Hr2Dx procedure into the urology workflow, in which patients are not currently expected to remain on-site after their biopsy procedures and time is not allocated for an immediate follow-up urologist consultation based on the 1Hr2Dx results.

## 5 Conclusions

In this study, we developed and demonstrated the feasibility and accuracy of a nondestructive procedure that relies on a 3D pathology platform to diagnose a set of 12 prostate needle cores within an hour of receipt. Such a method could potentially provide patients and their health care providers with a preliminary on-site diagnosis after a biopsy procedure, thereby alleviating anxiety and potentially expediting treatments.

## Code, Data, and Materials Availability

Deidentified prostate biopsy data may be available under a material transfer agreement. Instrumentation codes and schematics are available from the authors upon request.
