# Supplementary material for: Diagnosing 12 prostate needle cores within an hour of biopsy via open-top light-sheet microscopy
Source: J Biomed Opt. 2020 Dec 15;25(12):126502. doi: 10.1117/1.JBO.25.12.126502 (PMC7744172; doi:10.1117/1.JBO.25.12.126502)
Supplement: Supplementary file 1 [file JBO_025_126502_SD001.pdf]

Appendix A: Supplemental Material

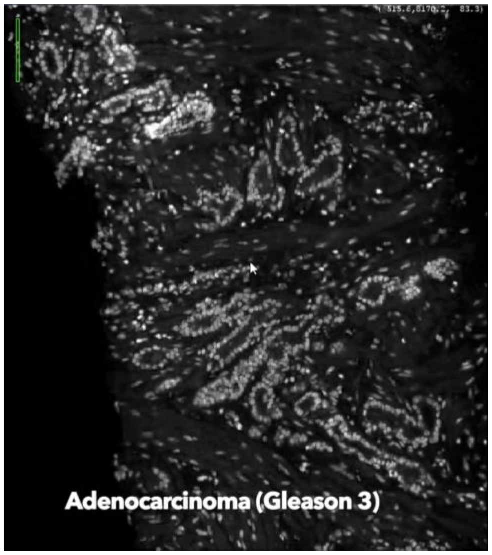

Video S1. Video of a pathologist viewing regions of a biopsy at different magnifications. In sequence, views are shown of benign glands, a focus of cancer glands, and finally, cancer glands admixed with benign glands. [MP4, 35MB]

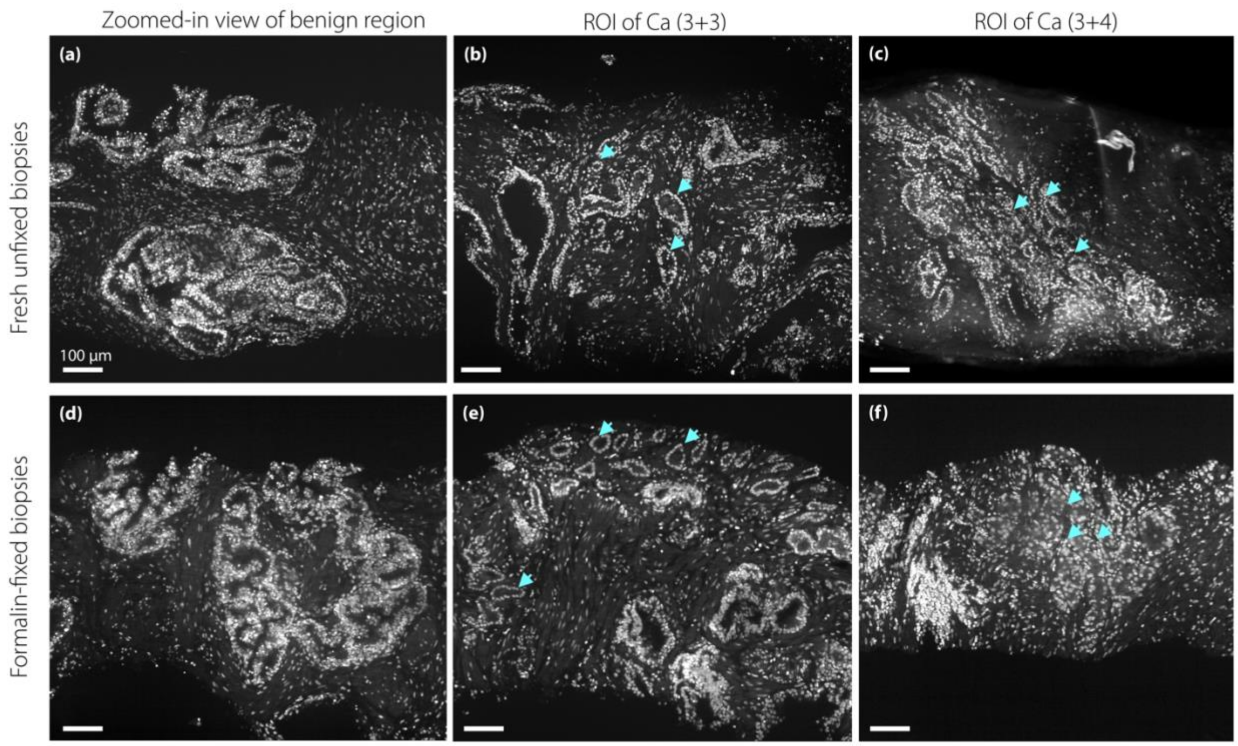

Figure S1. Images of fresh unfixed biopsies (a-c) with paired images of formalin-fixed biopsies (d-e). Examples are shown of benign glands (a, d), well-formed Gleason pattern 3 adenocarcinoma (arrows, b, e), and fused Gleason pattern 4 adenocarcinoma (arrows c, f). The quality of the paired images is similar.

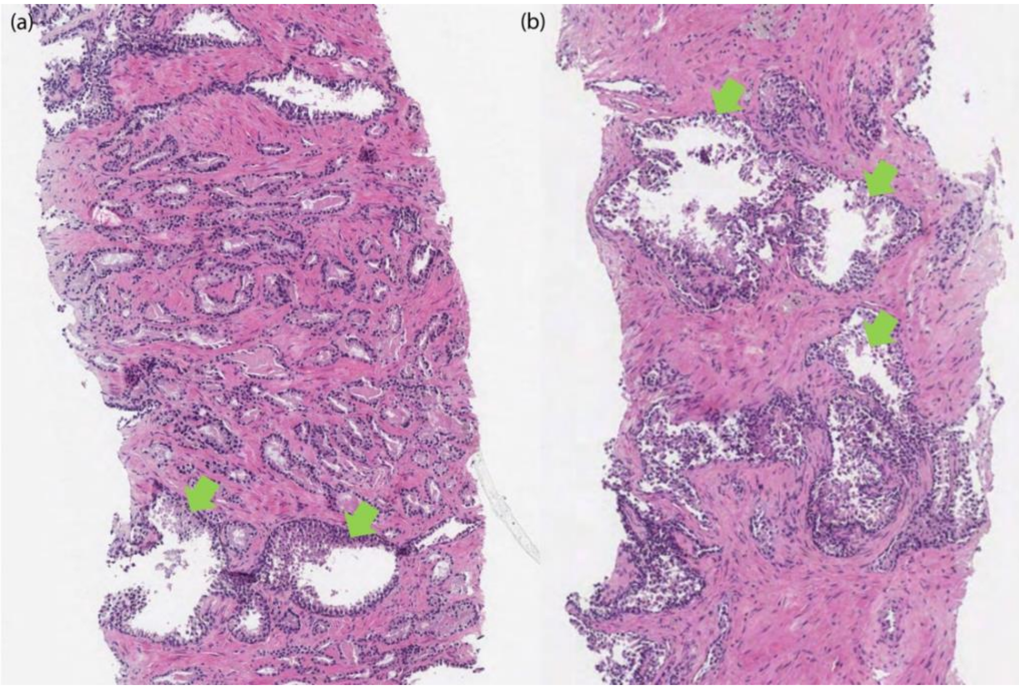

Figure S2. The 1Hr2Dx procedure was performed on fresh unfixed biopsies. The biopsies were subsequently fixed in formalin and processed for routine histology (H&E stain). (a-b) Conventional slide-based H&E histology images are shown of these specimens (after 1Hr2Dx). Note that detachment of some epithelial cells is seen, especially in benign glands (green arrows).

H&E Gleason grade for 10 cancerous biopsies (majority opinion)	Length of cancer (cm)	Length of biopsy (cm)	Cancer as % of length
3+3	0.45	1.50	30.0%
3+3	0.12	1.20	10.0%
3+3	0.30	1.20	25.0%
3+4	0.25	1.10	22.7%
3+3	0.20	0.90	22.2%
3+3	0.65	1.00	65.0%
3+3	0.55	1.30	42.3%
3+3	0.10	1.60	6.3%
3+3	0.70	1.30	53.8%
3+3	0.09	0.75	12.0%

Table S1. Characteristics of the 10 cancerous biopsies based on 2D H&E slides. Gleason grades and cancer length ratios were assigned to each biopsy retrospectively by 3 pathologists after the pilot study.
